# Rapid differentiation of MOGAD and MS after a single optic neuritis

**DOI:** 10.1007/s00415-024-12666-w

**Published:** 2024-09-09

**Authors:** T. Pakeerathan, J. Havla, C. Schwake, A. Salmen, M. Ringelstein, O. Aktas, M. Weise, J. A. Gernert, B. Kornek, G. Bsteh, A.-K. Pröbstel, A. Papadopoulou, L. Kulsvehagen, A. B. Ayroza Galvão Ribeiro Gomes, N. Cerdá-Fuertes, F. C. Oertel, A. S. Duchow, F. Paul, J. P. Stellmann, N. Stolowy, K. Hellwig, C. Schneider-Gold, T. Kümpfel, R. Gold, P. Albrecht, I. Ayzenberg

**Affiliations:** 1 Department of Neurology, St. Josef-Hospital, Ruhr-University Bochum, Gudrunstr. 56, 44791 Bochum, Germany; 2 Institute of Clinical Neuroimmunology, LMU Hospital, Ludwig-Maximilians Universität München, Munich, Germany; 3 Department of Neurology, Medical Faculty, Heinrich-Heine-University Düsseldorf, Düsseldorf, Germany; 4 Department of Neurology, Center for Neurology and Neuropsychiatry, LVR-Klinikum, Heinrich-Heine-University Düsseldorf, Düsseldorf, Germany; 5 Department of Neurology, Medical University of Vienna, Vienna, Austria; 6 Comprehensive Center for Clinical Neurosciences and Mental Health, Medical University of Vienna, Vienna, Austria; 7 Department of Neurology, University Hospital Basel and University of Basel, Basel, Switzerland; 8 Department of Biomedicine and Clinical Research, University Hospital Basel and University of Basel, Basel, Switzerland; 9 Research Center of Clinical Neuroimmunology and Neuroscience Basel (RC2NB), University Hospital Basel and University of Basel, Basel, Switzerland; 10 Translational Imaging in Neurology (ThINk) Basel, Department of Biomedical Engineering, University Hospital Basel and University of Basel, Basel, Switzerland; 11 Neuroscience Clinical Research Center, Charité-Universitätsmedizin Berlin, corporate member of Freie Universität Berlin and Humboldt-Universität zu Berlin, Berlin, Germany; 12 Experimental and Clinical Research Center, Max-Delbrück-Centrum für Molekulare Medizin and Charité-Universitätsmedizin Berlin, corporate member of Freie Universität Berlin and Humboldt-Universität zu Berlin, Berlin, Germany; 13 APHM, Hopital de La Timone, CEMEREM, Marseille, France; 14 Aix Marseille Univ, CNRS, CRMBM, Marseille, France; 15 Department of Ophthalmology, Centre Hospitalier Universitaire de La Timone, Marseille, France; 16 Department of Neurology, Kliniken Maria Hilf Mönchengladbach, Mönchengladbach, Germany

**Keywords:** Optical coherence tomography, Optic neuritis, Visual evoked potential, Myelin-oligodendrocyte-glycoprotein IgG, Myelin oligodendrocyte glycoprotein IgG-associated disease, Multiple sclerosis

## Abstract

**Background:**

Optic neuritis (ON) is a common manifestation of multiple sclerosis (MS) and myelin-oligodendrocyte-glycoprotein IgG-associated disease (MOGAD). This study evaluated the applicability of optical coherence tomography (OCT) for differentiating between both diseases in two independent cohorts.

**Methods:**

One hundred sixty two patients from seven sites underwent standard OCT and high-contrast visual acuity (HCVA) testing at least 6 months after first ON. Of these, 100 patients (32 MOGAD, 68 MS) comprised the primary investigational cohort, while 62 patients (31 MOGAD, 31 MS) formed a validation cohort. A composite score distinguishing between MOGAD and MS was developed using multivariate logistic regression.

**Results:**

Bilateral simultaneous ON occurred more frequently in MOGAD compared to MS (46.9 vs. 11.8%, *p* < 0.001). OCT revealed more peripapillary retinal nerve fiber layer (pRNFL) atrophy in all segments in MOGAD compared to predominantly temporal pRNFL atrophy in MS (*p* < 0.001). HCVA was better preserved in MS (*p* = 0.007). pRNFL thickness in all except for temporal segments was suitable for differentiating MOGAD and MS. Simultaneous bilateral ON and critical atrophy in nasal (< 58.5 µm) and temporal superior (< 105.5 µm) segments were included into the composite score as three independent predictors for MOGAD. The composite score distinguished MOGAD from MS with 75% sensitivity and 90% specificity in the investigational cohort, and 68% sensitivity and 87% specificity in the validation cohort.

**Conclusion:**

Following a single ON-episode, MOGAD exhibits more pronounced global pRNFL atrophy and lower visual acuity after ON compared to MS. The introduced OCT-based composite score enabled differentiation between the two entities across both cohorts.

## Introduction

Optic neuritis (ON) is one of the major manifestations of multiple sclerosis (MS) and myelin-oligodendrocyte glycoprotein (MOG) immunoglobulin G-associated disease (MOGAD) [1–3]. Approximately 70% of MS and 54–61% of MOGAD patients experience ON during the course of their disease [1, 2]. MOGAD can be monophasic, still in 50% of patients relapses can be observed [4, 5]. Relapsing ON may substantially influence the clinical outcome, although MOGAD patients usually show a good visual recovery after ON [6]. Bilateral ON manifestation occurs more frequently in MOGAD patients in comparison to MS patients [2, 5]. Due to the overlapping clinical manifestations distinguishing between the two entities can be challenging. Banwell et al. proposed diagnostic criteria for MOGAD recommending that MS must be excluded to diagnose MOGAD [3]. A correct diagnosis is of high importance as the pathophysiological mechanisms differ and classical MS drugs may be ineffective or even worsen the course of MOGAD [7].

The presence of conformation-dependent autoantibodies against MOG is one of the main requirements for fulfillment of the MOGAD diagnostic criteria [3, 7, 8]. However, borderline serum titers of MOG-immunoglobulin G (IgG) have a low-positive predictive value and can be found in other neurologic diseases, including MS. Seroreversion may occur in the first months after MOGAD onset [8, 9]. Several groups recently reported presence of isolated MOG-IgA in serum or isolated intrathecal production of MOG-IgG in 12–13% patients, accordingly requiring specific tests or invasive diagnostic procedures [10–12]. Considering the limitations and the unavailability of appropriate live cell-based assays (CBA) for MOG-IgG in many countries, an additional paraclinical diagnostic marker for MOGAD may be useful in daily clinical practice, especially in case of borderline serum titer of MOG-IgG.

Optical coherence tomography (OCT) allows precise assessment of retinal neuroaxonal atrophy. Although peripapillary retinal nerve fiber layer (pRNFL) thickening is sensitive in differentiating MOGAD and MS during the acute ON, there are only a few studies evaluating diagnostic accuracy of OCT in the chronic ON stage in adult patients after the first ON episode [13, 14]. Preliminary results from a small pediatric cohort, published by our group previously, suggested different atrophy patterns in MS and MOGAD [15].

Main objectives of this study were: (1) to examine the distinct pattern of retinal neuroaxonal atrophy in MOGAD and MS, (2) to analyze the sensitivity and specificity of OCT in distinguishing between MOGAD and MS, and (3) to compare visual outcomes in both diseases after the first ON episode.

## Subjects and methods

**Fig. 1:**
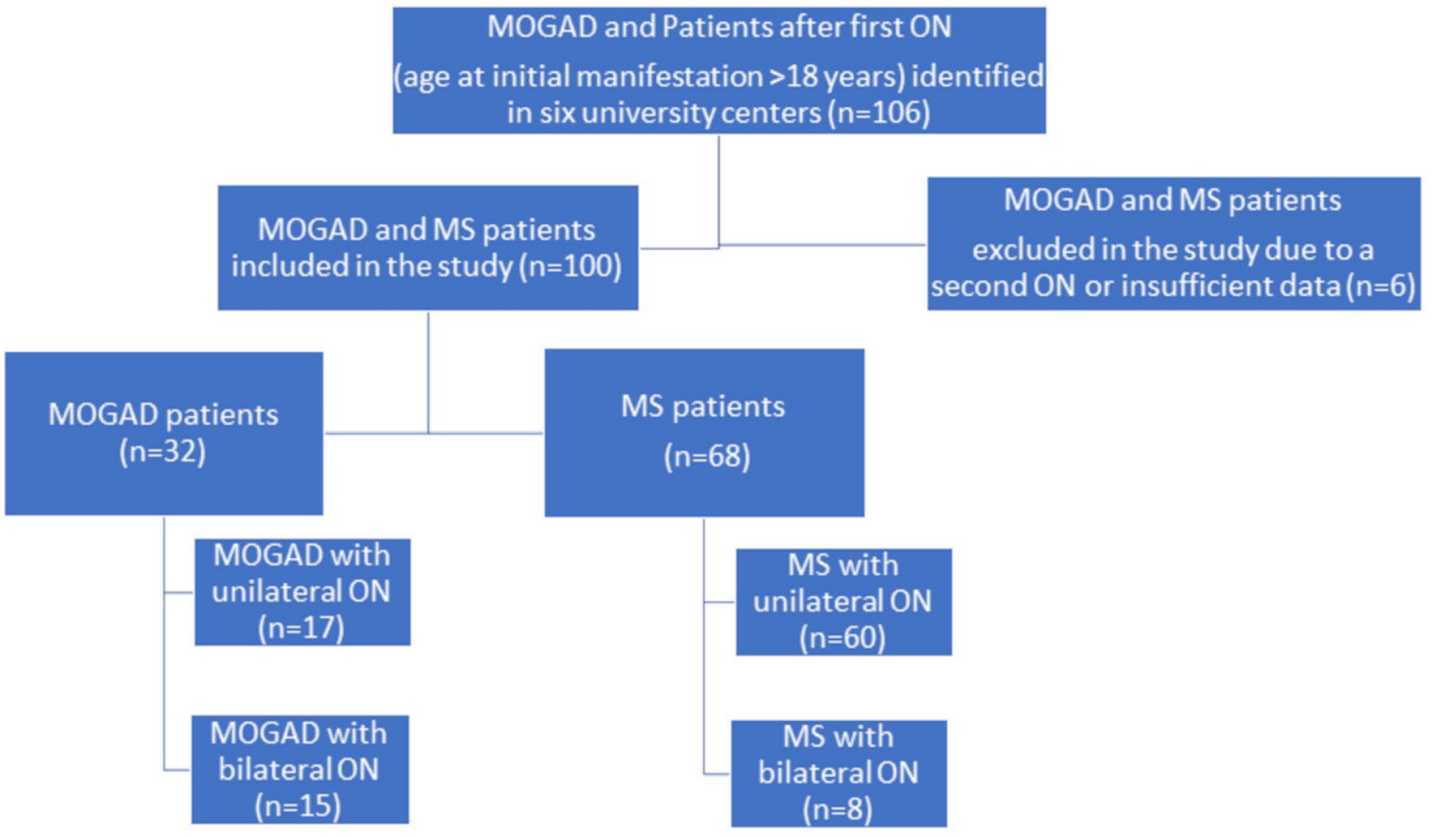
Flow chart of patients included in the investigational cohort. We included 100 MOGAD or MS patients after the first ON episode who were identified in the participating centers. Depending on the diagnosis the patients were divided into two groups: group (1) 32 MOG-IgG-patients with initial manifestation > 18 years and group (2) 68 MS patients with initial manifestation > 18 years

We conducted a multicenter, retrospective cross-sectional study, comparing clinical and OCT data of MOGAD and MS patients after a single ON episode, fulfilling the following inclusion criteria: (1) MOG-IgG positive status (> 1:100 in fixed or > 1:320 in live CBA) or diagnosis of MS according to McDonald criteria 2017 [16]; (2) age at first ON episode > 18 years; (3) OCT and HCVA examinations were performed at least 6 months after the first ON episode. The exclusion criteria for this study were: (1) patients with concomitant ophthalmological diseases; (2) patients seropositive for aquaporin-4 IgG; (3) recurrent ON before enrollment. The investigational cohort was recruited between 2018 and 2022 at six university tertiary care centers specialized in neuroimmunology (Munich, Düsseldorf, Vienna, Basel, Berlin, Bochum, Fig. 1, study participants). After the MOGAD Banwell criteria were published we re-evaluated clinical data of included patients in the investigational cohort. The majority (85%) fulfilled the new diagnostic criteria, while data was insufficient or missing for 15% of the patients. The validation cohort was recruited in 2023 at five university tertiary care centers (Munich, Düsseldorf, Marseille, Berlin, Bochum). During the initial workup patients´ serum samples were tested for MOG-IgG and aquaporin4-IgG at least once by established CBA at the discretion of each center using the laboratories´ cut-offs (MOG IFT, EUROIMMUN, Laboratory Prof. Stöcker, Germany; Laboratory Prof. Reindl, Medical University of Innsbruck, Innsbruck, Austria; Laboratory Prof. Meinl, LMU Hospital, Munich, Germany) [7, 17].

We acquired demographic and clinical data for all MOGAD and MS patients. Diagnosis of unilateral or bilateral ON manifestation were based on the clinical history. In addition, we obtained monocular high contrast visual acuity (HCVA) using standardized retro-illuminated Sloan letter charts (maximum: 70 letters). HCVA data is only available for the investigational cohort. Ethics approval was obtained in the participating centers respectively. All patients gave written informed consent for scientific analysis. The study was conducted according to the Declaration of Helsinki (1964) in its currently applicable version.

### Optical coherence tomography (OCT)

Spectral-domain optical coherence tomography (SD-OCT, SPECTRALIS, Heidelberg Engineering, Heidelberg, Germany) with automatic real-time (ART) averaging was uniformly utilized across all participating centers. A ring scan of the optic nerve head with an activated eye tracker (12°, 3,5 mm ring, 50 ≤ ART ≤ 100) and a macular volume scan (20° × 20°, 25 vertical B-scans, 20 ≤ ART ≤ 49) with a grid as a fovea-centered cylinder of 3 mm diameter were conducted based on local protocols. The pRNFL thickness and the volumes of the macular retinal nerve fiber layer (mRNFL), the combined ganglion cell and inner plexiform layer (GCIP), the inner nuclear layer (INL), the combined outer plexiform and outer nuclear layer (ONPL) and the total macular volume (TMV) were included in the analysis. The segmentation of all layers was conducted semi-automatically using the software of the SD-OCT manufacturer [Heidelberg Eye Explorer (HEYEX) 1.9.10.0 with viewing module 6.3.4.0, Heidelberg Engineering, Heidelberg, Germany]. Experienced evaluators carefully checked all scans for sufficient quality as well as segmentation errors, which were corrected manually if necessary. The SD-OCT data were analyzed and reported according to the recommendations of APOSTEL2.0 and OSCAR-IB [18, 19].

### Statistical methods

Clinical, OCT, and HCVA data were compared between MOGAD and MS_._ For continuous variables mean and standard deviation (SD) were calculated, for categorical variables frequency and proportion. The non-parametric Mann-Whitney-*U*-Test and Chi-Square-Test were used to compare two independent groups. Statistical significance was defined as *p* < 0.05. We outlined frequencies of significant atrophy in different pRNFL quadrants to illustrate the pattern of retinal changes after a single ON. We also reported frequencies of severe atrophy in different pRNFL segments and macular sectors in both groups, defined as a decrease of two SDs below the mean reported by Heidelberg Engineering based on data from healthy controls, compared to the standard values of Heidelberg Engineering. OCT and HCVA data in ON eyes were compared between the MOGAD and MS cohorts using generalized estimating equation models (GEE) to account for within-patient inter-eye correlations. The correlation-matrix parameter was set to “exchangeable”. Statistically significantly different (*p* < 0.05) parameters were further included into Receiver Operating Characteristic (ROC) analysis to determine their sensitivity and specificity in differentiating MOGAD from MS. Independent parameters with an area under the curve (AUC) > 0.7 were reported and considered as suitable parameters to differentiate between the two entities. To determine optimal cut-off values, we used the Youden index. To formulate a clinically relevant composite score, a multivariate logistic regression model was fitted, incorporating age, sex, and the most appropriate clinical and OCT-parameters for distinguishing between MOGAD-ON and MS-ON. The model was reduced based on Akaike information criterion with a stepwise selection of variables. Data were analyzed with SPSS version 29 (IBM SPSS Statistics) and Statics in R. We used STROBE cross sectional reporting guidelines to report the study data [20].

## Results

### Investigational cohort

Thirty-two MOGAD [female:male 19:13, age at ON (mean ± SD: 35.3 ± 11.7 years), 47 ON eyes] and 68 MS [female: male 52:16, age at ON (mean ± SD 33.1 ± 10.9 years), 76 ON eyes] patients with a history of a single unilateral or bilateral ON episode were included in the investigational cohort. The main demographic and clinical data of both groups are summarized in Table 1. All MOGAD patients were tested negative for cerebrospinal fluid-specific oligoclonal bands (OCB), and serum aquaporin-4 IgG. All patients in the MS cohort presented cerebrospinal fluid-specific OCBs. The age at ON and disease duration were comparable between both groups. ON was the initial disease manifestation in 71.8% of MOGAD and 57.4% of MS patients. Simultaneous bilateral ON was more prevalent in MOGAD compared to MS patients (46.9 vs. 11.8%, *p* < 0.001). Nine MOGAD-ON (19.1%) and two MS-ON (2.6%) required plasma exchange due to steroid refractory ON (*p* = 0.004). HCVA was significantly lower in MOGAD compared to MS at least six months after ON manifestation (49.2 ± 14.4 vs. 54.2 ± 11.4 letters, *p* = 0.007). A long-term immunotherapy was administered in 20 of 32 (62.5%) of MOGAD and 50 of 68 (73.5%) of MS patients.

**Table 1:** Demographic and main clinical characteristics of MOGAD and MS patients

	Investigational cohort			Validation cohort		
	MOGAD (*n* = 32)	MS (*n* = 68)	*p* value	MOGAD (*n* = 31)	MS (*n* = 31)	*p* value
Age at ON in years, mean ± SD	35.3 ± 11.7	33.1 ± 10.9	0.160	38.6 ± 15.9	36.6 ± 12.5	0.578
Females, *n* (%)	**19 (59.4%)**	**52 (76.5%)**	**0.013**	19 (61.3%)	19 (61.3%)	1
Time interval ON onset and OCT examination in months, mean ± SD	**24.3 ± 28.9**	**30.2 ± 24.5**	**0.014**	71.8 ± 236.9	41.9 ± 48.6	0.072
Disease duration (in years), mean ± SD	3.7 ± 4.4	4.9 ± 14.7	0.742	–	–	–
Patients with simultaneous bilateral ON, *n* (%)	**15 (46.9%)**	**8 (11.8%)**	**< 0.001**	**14 (45.2%)**	**1 (3.2%)**	**0.001**
Total ON eyes, *n* (%)	**47 (73.4%)**	**76 (55.9%)**	**0.017**	45 (72.6%)	32 (51.6%)	0.28
Positive OCB, *n* (%)	0 (0%)	68 (100%)	–	–	–	–
HCVA, number of correctly stated letters, mean ± SD	**49.2 ± 14.4**	**54.2 ± 11.4**	**0.007**	–	–	–

*MOGAD* myelin-oligodendrocyte glycoprotein IgG-associated disease, *MS* multiple sclerosis, *ON* optic neuritis, *OCB* oligoclonal bands, *HCVA* high contrast visual acuity, *SD* standard deviation, significant results *p* < 0.05 are indicated in bold letters

### Peripapillary and macular retinal atrophy patterns in MOGAD and MS

**Fig. 2:**
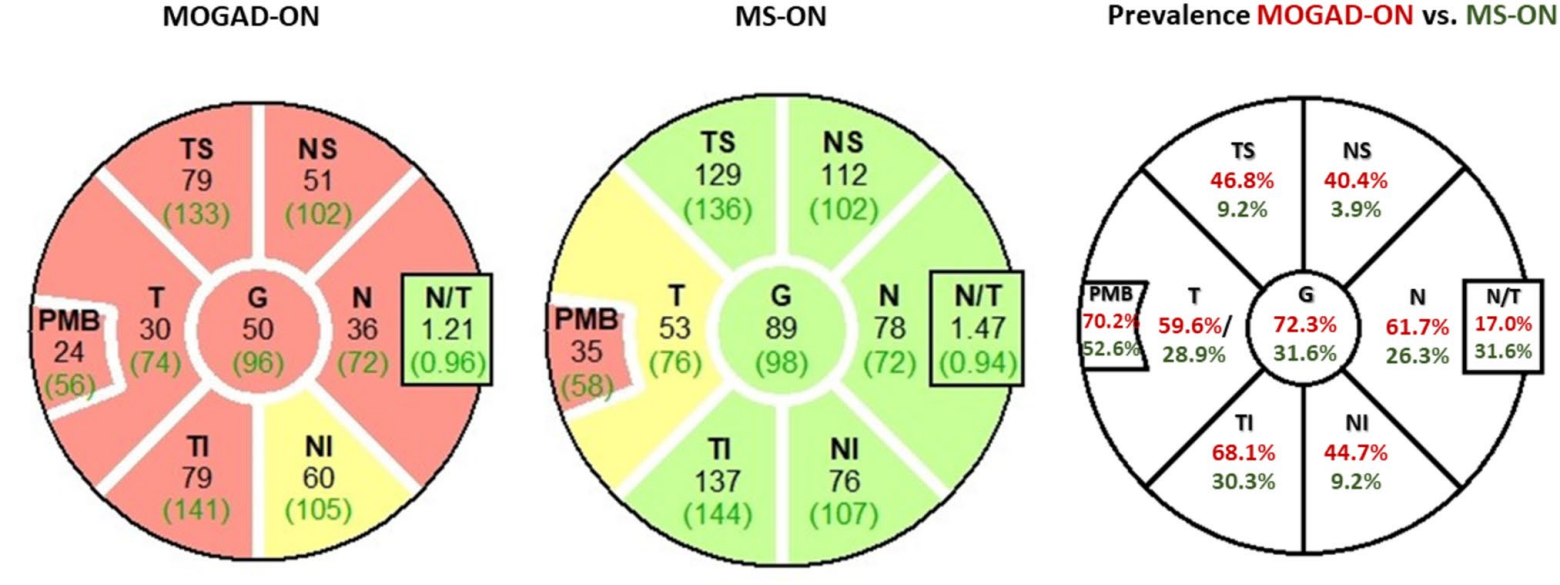
Exemplary OCT ringscans and prevalence of pathological results in MOGAD and MS patients. Exemplary OCT ringscans show the typical atrophy patterns in MOGAD and MS patients after ON with a predominantly temporal pRNFL thinning in MS patients compared to the global retinal atrophy in MOGAD patients. The prevalence of pathological results, two standard deviations below the mean based on the data from healthy cohorts reported by Heidelberg Engineering, is visually represented in the figure for MOGAD-ON (in red letters) and MS-ON (in green letters)

The OCT measures and prevalence of pRNFL-atrophy are outlined in Table 2 and Fig. 2. We observed a substantial difference in the patterns of peripapillary retinal axonal degeneration between MOGAD-ON and MS-ON eyes in the chronic stage. There was a notably more pronounced global and segmental atrophy in MOGAD-ON, while MS-ON demonstrated predominantly temporal moderate pRNFL thinning. The pRNFL thickness in the papillomacular bundle (PMB) was comparable between groups. The prevalence of severe pRNFL atrophy, when compared to normal range data reported by Heidelberg Engineering, were significantly different both globally (MOGAD: 72.3% vs. MS: 31.6%) as well as in all segments. In addition, there was a more pronounced reduction in the thickness of the macular RNFL and GCIP in MOGAD-ON compared to MS-ON eyes (mRNFL: *p* = 0.033, GCIP: *p* = 0.018, Table 2).

**Table 2:** OCT measures after a single ON in MOGAD and MS (investigational cohort)

Parameter	MOGAD ON-eyes (*N* = 47, mean ± SD)	MS ON-eyes (*N* = 76, mean ± SD)	MOGAD vs. MS,* p *value	Cut-off value (mean-2 SD)	MOGADON-eyes (*N* = 47, number of eyes with severe atrophy)	MS ON-eyes (*N* = 76, number of eyes with severe atrophy)
G pRNFL	**70.5 ± 18.9**	**86.1 ± 14.7**	**< 0.001**	80.6 µm	34 (72.3%)	24 (31.6%)
T pRNFL	**48.7 ± 15.2**	**55.6 ± 13.8**	**0.031**	50.9 µm	28 (59.6%)	22 (28.9%)
TS pRNFL	**102.1 ± 27.5**	**121.1 ± 21.8**	**< 0.001**	88.8 µm	22 (46.8%)	7 (9.2%)
TI pRNFL	**103.5 ± 28.3**	**126.0 ± 27.2**	**< 0.001**	115 µm	32 (68.1%)	23 (30.3%)
N pRNFL	**50.8 ± 16.1**	**65.1 ± 13.9**	**< 0.001**	56.5 µm	29 (61.7%)	20 (26.3%)
NS pRNFL	**77.3 ± 25.9**	**100.2 ± 20.0**	**< 0.001**	67.2 µm	19 (40.4%)	3 (3.9%)
NI pRNFL	**82.9 ± 32.8**	**103.0 ± 26.2**	**0.002**	66.5 µm	21 (44.7%)	7 (9.2%)
PMB	39.4 ± 11.3	42.3 ± 11.4	0.232	44.6 µm	33 (70.2%)	40 (52.6%)
N/T Ratio	**1.06 ± 0.32**	**1.2 ± 0.4**	**0.006**	0.56 < x < 1.36	8 (17.0%)	24 (31.6%)
TMV 3 mm	**2.15 ± 0.21**	**2.25 ± 0.12**	**0.015**			
mRNFL 3 mm	**0.13 ± 0.02**	**0.14 ± 0.02**	**0.033**			
GCIP 3 mm	**0.46 ± 0.11**	**0.51 ± 0.1**	**0.018**			
INL 3 mm	0.27 ± 0.03	0.27 ± 0.02	0.409			
ONPL 3 mm	0.74 ± 0.05	0.75 ± 0.04	0.360			

The table compares the mean pRNFL thickness as well as the prevalence of severe pRNFL-atrophy in MOGAD and MS after a single ON

*MOGAD* myelin-oligodendrocyte-glycoprotein IgG-associated disease, *MS* multiple sclerosis, *ON* optic neuritis, *MOGAD-ON* MOGAD patient’s eyes with a history of ON, *MS-ON* MS patient’s eyes with a history of ON,* pRNFL* peripapillary retinal nerve fiber layer (*G* global, *T* temporal, *TS* temporal superior, *TI* temporal inferior, *N* nasal, *NS* nasal superior, *NI* nasal inferior, *PMB* papillomacular bundle, *N/T* nasal/temporal ratio), *TMV* total macular volume, *mRNFL* macular retinal nerve fiber layer, *mGCIP* macular ganglion cell and inner plexiform layer, *mINL* macular inner nuclear layer, *mONPL* macular outer plexiform and outer nuclear layer. pRNFL thickness in µm and macular volumes in mm^3^. *p* value: significant results *p* < 0.05 are indicated in bold letters

### Composite score enables differentiation between MOGAD and MS

All independent OCT-parameters that showed significant differences between groups were included in the ROC analysis. In addition, we considered bilateral eye involvement as a highly relevant clinical parameter. Comparison of all ON-eyes revealed that all pRNFL segments except for the temporal segment enabled the distinction of MOGAD from MS (AUC > 0.7). Neither the macular layers nor visual acuity allowed differentiation between the groups.

In a more in-depth analysis, we included only one ON-eye per patient, choosing the eye with the more severe global pRNFL atrophy in case of bilateral involvement. This refinement resulted in increased AUC-values, sensitivity, and specificity accordingly (s. Table 3).

**Table 3:** Sensitivity and specificity of pRNFL parameters in distinguishing MOGAD-ON and MS-ON eyes (investigational cohort)

Parameter/segment	All ON eyes				One eye per patient			
	AUC	Cut-off	Sensitivity for MOGAD vs. MS	Specificity for MOGAD vs. MS	AUC	Cut-off	Sensitivity for MOGAD vs. MS	Specificity for MOGAD vs. MS
pRNFL G	0.740	75.6 µm	66.0%	78.9%	0.781	75.6 µm	71.9%	80.9%
pRNFL N	0.746	58.5 µm	68.1%	73.7%	**0.787**	**58.5 µm**	**75.0%**	**73.5%**
pRNFL NS	0.759	78.0 µm	59.6%	86.8%	0.763	78.0 µm	56.3%	89.7%
pRNFL NI	0.706	73.0 µm	53.2%	88.2%	0.741	73.0 µm	59.4%	89.7%
pRNFL TS	0.706	105.5 µm	61.7%	82.9%	**0.759**	**105.5 µm**	**68.8%**	**83.8%**
pRNFL TI	0.718	114.5 µm	68.1%	71.1%	0.767	114.5 µm	75.0%	70.6%

Only parameters with AUC > 0.700 were considered as suitable parameters which are listed in the table. Parameters with the highest sensitivity and specificity are indicated in bold letters. Only one ON-eye per patient was included in the subgroup analysis. In case of bilateral ON, the ON eye with the worse global pRNFL atrophy was chosen

*MOGAD* myelin-oligodendrocyte-glycoprotein -antibody-associated disease, *MS* multiple sclerosis, *ON* optic neuritis, *MOGAD-ON* MOGAD patient’s eyes with a history of ON, *MS-ON* MS patient’s eyes with a history of ON, *pRNFL* peripapillary retinal nerve fiber layer (*G* global, *TS* temporal superior, *TI* temporal inferior, *N* nasal, *NS* nasal superior, *NI* nasal inferior), *AUC* area under the curve. pRNFL thickness in µm

**Fig. 3:**
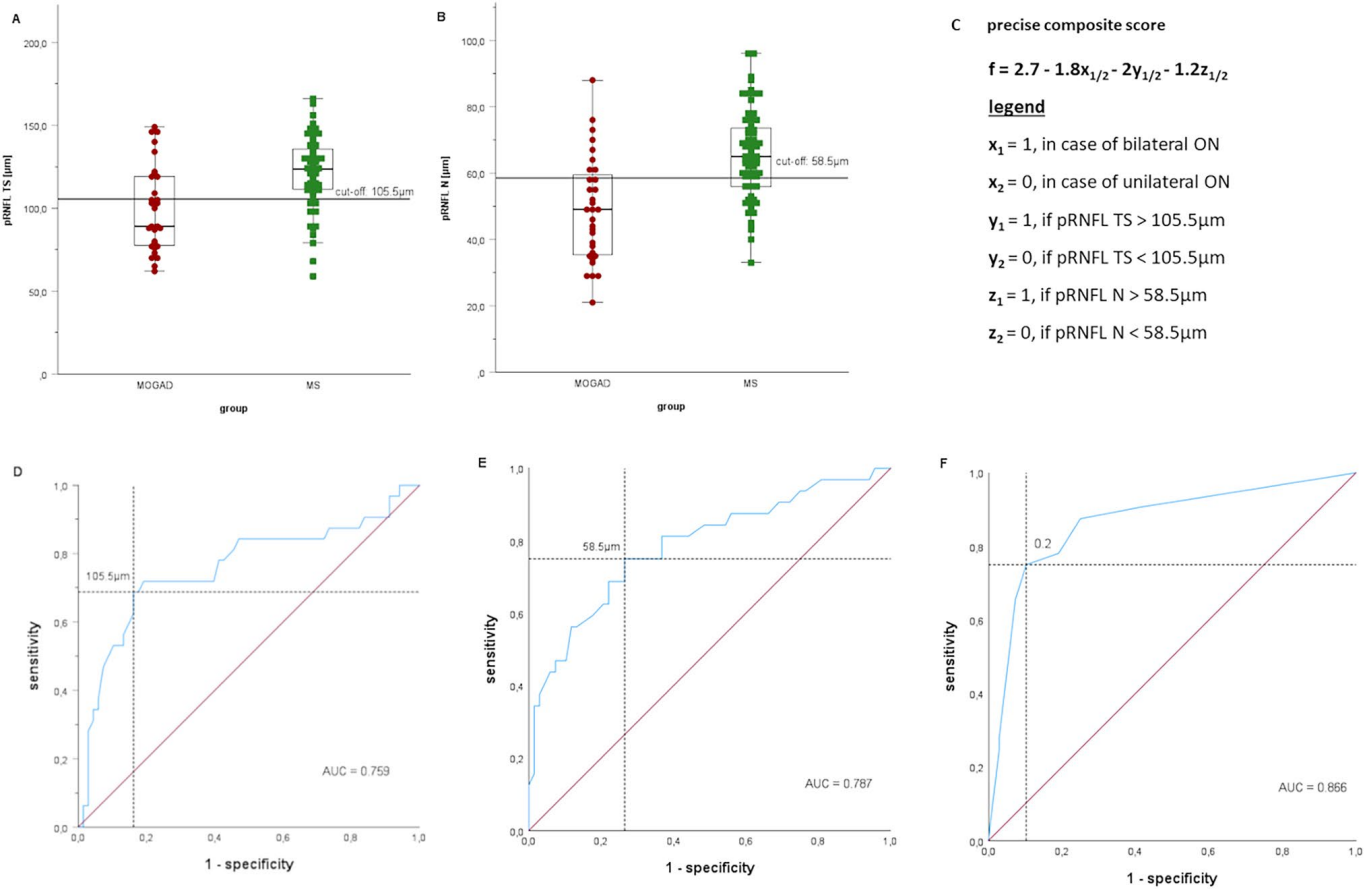
pRNFL thickness and precise composite score in MOGAD-ON and MS-ON. Figure 3 consists of diagrams, visualizing the distribution of **A** temporal superior pRNFL thickness **B** nasal pRNFL thickness in MOGAD-ON and MS-ON. Further the figure shows **C** the formula for the precise composite score, the ROC curves with the cut-offs for **D** pRNFL TS, **E** pRNFL N and **F** the precise composite score

To achieve the highest diagnostic accuracy, we built a composite score, based on the logistic regression model including sex, age at ON, bilateral ON (yes/no) and critical pRNFL atrophy (yes/no, s. Table 3 for cut-offs) for the temporal superior, temporal inferior, nasal superior, nasal and nasal inferior segments in one eye per patient. Atrophy in nasal and temporal superior segments as well as bilateral involvement were three independent predictors (s. Figure 3c). The composite score enabled distinguishing MOGAD and MS patients with a higher accuracy in comparison to the individual segments (AUC = 0.866) reaching a sensitivity of 75% and a specificity of 89.7% (s. Figure 3f). To construct a simplified score for clinical use we rounded the linear estimates from the regression model without relevant effect on the performance and established a score ranging from 0 to 5 (s. Table 4). The positive predictive value of the simplified score was 80%.

**Table 4:** Sensitivity and specificity of the simplified composite score in distinguishing MOGAD-ON and MS-ON patients

	Investigational cohort		Validation cohort	
Cut-off	Sensitivity	Specificity	Sensitivity	Specificity
≥ 0	100%	0%	100%	0%
≥ 1	90.6%	58.8%	83.9%	54.8%
≥ 2	87.5%	75%	74.2%	71.0%
**≥ 3**	**75%**	**89.7%**	**67.7%**	**87.1%**
≥ 4	28.1%	97.1%	32.3%	100%
5	25.0%	97.1%	25.8%	100%

Composite score, consisting of the three strongest parameters (bilateral ON, pRNFL atrophy in temporal superior and nasal segments), enables differentiation between MOGAD and MS patients with a single ON episode with high accuracy. Bilateral ON and temporal superior pRNFL thickness below 105.5 µm each equal two points. Nasal pRNFL thickness below 58.5 µm equals one point. Only one ON-eye per patient was included in the sub analysis. In case of bilateral ON, the ON eye with the higher global atrophy was included, most suitable cut-off indicated in bold letters

### Validation of the composite score in an independent cohort

In an independent validation cohort we included 31 MOGAD patients (female:male 19:12) with 45 ON eyes and 31 MS patients (female:male 19:12) with 32 ON eyes (s. Table 1). The mean age at ON in MOGAD patients was 38.6 ± 15.9 years, whereas in MS patients the mean age at ON was 36.6 ± 12.5 years (*p* = 0.578). Bilateral ON occurred more frequently in MOGAD than MS (MOGAD: 45.2% of and MS: 3.2%, *p* < 0.001). The time interval between ON and OCT was not significantly different with 71.78 ± 236.9 months in MOGAD and 41.9 ± 48.6 months in MS patients (*p* = 0.072). The accuracy of the precise composite score could be confirmed in the validation cohort at a cut-off of 0.2 with 67.7% sensitivity and 87.1% specificity. In a simplified composite score we could also demonstrate a sensitivity of 67.7% as well as specificity of 87.1% for a cut-off of 3 points (s. Table 4).

## Discussion

In this study, we compared retinal atrophy patterns and visual outcomes at least 6 months after the first ON episode in MOGAD and MS patients and evaluated the accuracy of OCT in distinguishing between both diseases. Similar to previous studies bilateral ON occurred significantly more often in MOGAD compared to MS patients (MOGAD 44–51% vs. MS 3–11%) [7–10, 21, 22]. The visual outcome was significantly better in MS than in MOGAD. In contrast, Akaishi et al. demonstrated that MOGAD- and MS-ON result in comparable visual acuity at nadir, 1 year as well as 5 years after ON [23]. The depicted difference can be probably explained by the different ethnic composition and substantially smaller sample size in the Japanese study. MOGAD patients showed significantly more pronounced global pRNFL atrophy compared to a typical moderate predominantly temporal retinal thinning of MS patients [24–30]. The RNFL thickness of all peripapillary segments was also lower in the MOGAD cohort. The detected differences in atrophy patterns can be explained by the different underlying mechanisms of both conditions. Primary CD8 + T-cell modulated inflammation, involving only short segments of the optic nerve, occurs in MS [5, 31]. Presumed secondary mitochondrial dysfunction and predominant demyelination of the most energy-dependent temporal fibers with a high firing rate may contribute to the foremost temporal retinal thinning. Especially smaller and thinly myelinated parvocellular axons of the PMB are known to be more vulnerable to oxidative stress in MS [25, 32, 33]. In contrast, an acute primary MOG-IgG/CD4 + T-cells-related longitudinal inflammation of the distal optic nerve causes a global perineural contrast enhancement with papilledema and equal affection of all ON fibers in MOGAD [14, 34, 35].

In contrast to pRNFL, the macular scan revealed only moderate differences in mRNFL and GCIP, not allowing differentiation between both conditions with sufficient accuracy, which corresponds well with observations made in an Australian cohort [31]. The active involvement of temporal pRNFL fibers in both diseases explains only moderate differences in macular atrophy in these conditions. Severe macular atrophy, demonstrated in some previous studies in MOGAD, is probably associated with a higher number of consecutive ON episodes [36].

The most striking and practically relevant result of our study is the composite score, consisting of the three following most suitable parameters: bilaterality of ON, temporal superior and nasal pRNFL thickness. The score allows the differentiation between MOGAD-ON and MS-ON with a 75% sensitivity and nearly 90% specificity. The score can be applied as a quick diagnostic tool, easy to perform in daily clinical practice. The accuracy of the score could be confirmed in an independent validation cohort. Compared to a previous study, demonstrating OCT-based differentiation between MOGAD and MS in the short acute phase (< 2 weeks after onset), our score can be used to distinguish between both entities in a chronic disease phase. As an additional paraclinical diagnostic marker it can be useful in selection of patients with ON in the history for MOG-IgG testing. Further studies are needed to evaluate diagnostic relevance of this score in differentiation between MOGAD and MS patients with a borderline serum MOG-IgG titer.

Our study has several limitations. We performed a retrospective analysis, therefore selection and reporting biases regarding ON and disease history cannot be excluded. Differences in disease duration between the investigational and validation cohorts is due to the rarity of MOGAD. Data on visual outcomes were available in the investigational cohort only and sample size were limited. Despite being able to differentiate between MOGAD and MS, this score is not helpful in distinguishing MOGAD-ON from other types of ON, including AQP4-IgG positive ON. The study was conducted before the MOGAD criteria were published; however, we were able to re-evaluate 85% of the patients, all of whom tested positive.

## Conclusion

In the current study, we report a markedly more pronounced global peripapillary retinal degeneration following an initial ON in MOGAD compared to MS. We developed an OCT-based composite score distinguishing between both diseases and confirmed its diagnostic accuracy in the independent validation cohort. Our study emphasizes the potential relevance of OCT as an accurate additional method in the diagnostic of MOGAD. MOG-IgG testing should be performed in all patients with a score of ≥ 1 following a history of one episode of ON. Further studies are needed to investigate the diagnostic relevance of this score in patients with borderline MOG-IgG titer.

## Declarations

### Data availability

The data that support the findings of this study are available from the corresponding author upon reasonable request.
